# Managing lower urinary tract symptoms in primary care: qualitative study of GPs’ and patients’ experiences

**DOI:** 10.3399/BJGP.2020.1043

**Published:** 2021-08-03

**Authors:** Sarah Milosevic, Natalie Joseph-Williams, Bethan Pell, Elizabeth Cain, Robyn Hackett, Ffion Murdoch, Haroon Ahmed, A Joy Allen, Alison Bray, Emma Thomas-Jones, Chris Harding, Adrian Edwards

**Affiliations:** Centre for Trials Research, Cardiff University, Cardiff.; PRIME Centre Wales, Division of Population Medicine, School of Medicine, Cardiff University, Cardiff.; Centre for the Development and Evaluation of Complex Interventions for Public Health Improvement (DECIPHer), Cardiff University, Cardiff.; PRIME Centre Wales, Division of Population Medicine, School of Medicine, Cardiff University, Cardiff.; PRIME Centre Wales, Division of Population Medicine, School of Medicine, Cardiff University, Cardiff.; PRIME Centre Wales, Division of Population Medicine, School of Medicine, Cardiff University, Cardiff.; PRIME Centre Wales, Division of Population Medicine, School of Medicine, Cardiff University, Cardiff.; National Institute for Health Research Newcastle In Vitro Diagnostics Co-operative, Newcastle University, Newcastle upon Tyne; Translational and Clinical Research Institute, Newcastle University, Newcastle upon Tyne.; Northern Medical Physics and Clinical Engineering, Newcastle upon Tyne Hospitals NHS Foundation Trust, Newcastle upon Tyne; honorary research associate, Translational and Clinical Research Institute, Newcastle University, Newcastle upon Tyne.; Centre for Trials Research, Cardiff University, Cardiff.; Department of Urology, Newcastle upon Tyne NHS Hospital Trust, Newcastle Freeman Hospital, Newcastle upon Tyne.; PRIME Centre Wales, Division of Population Medicine, School of Medicine, Cardiff University, Cardiff.

**Keywords:** general practice, lower urinary tract symptoms, primary health care, qualitative research, urology

## Abstract

**Background:**

Lower urinary tract symptoms (LUTS) are common in males aged ≥40 years and have a considerable impact on quality of life. Management can be complex, and although most LUTS could be treated effectively in primary care, referrals to urology outpatients are increasing.

**Aim:**

To explore GPs’ experiences of managing LUTS together with patients’ experiences of and preferences for treatment in primary care.

**Design and setting:**

Telephone interviews were conducted with GPs and male patients presenting to primary care with bothersome LUTS.

**Method:**

Eleven GPs and 25 male patients were purposively sampled from 20 GP practices in three UK regions: Newcastle upon Tyne, Bristol, and South Wales. Interviews were conducted between May 2018 and January 2019, and were analysed using a framework approach.

**Results:**

Difficulty establishing causes and differentiating between prostate and bladder symptoms were key challenges to the diagnosis of LUTS in primary care, often making treatment a process of trial and error. Pharmacological treatments were commonly ineffective and often caused side effects. Despite this, patients were generally satisfied with GP consultations and expressed a preference for treatment in primary care.

**Conclusion:**

Managing LUTS in primary care is a more accessible option for patients. Given the challenges of LUTS diagnosis, an effective diagnostic tool for use by GPs would be beneficial. Ensuring bothersome LUTS are not dismissed as a normal part of ageing is essential in improving patients’ quality of life. Greater exploration of the role of non-pharmacological treatments is needed.

## INTRODUCTION

It is estimated that 44.1% of males aged ≥20 years worldwide are affected by ≥1 lower urinary tract symptom (LUTS), a proportion projected to increase as the population ages.^1^^,^^2^ LUTS are associated with increased rates of anxiety and depression,^3^ and have an impact on work productivity, enjoyment of sexual activity, and overall health.^4^ Despite this, only a minority of males with LUTS consult their GP about their symptoms,^5^^,^^6^ and even fewer receive treatment.^7^^,^^8^

The frequent comorbidity of male LUTS with prostatic disease, together with a commonly multifactorial aetiology,^9^ means that diagnosis and management can be complex. In addition, GPs encounter new presentations of LUTS relatively infrequently, limiting their opportunity to develop expertise in this area. Therefore, although most males presenting with LUTS could be treated effectively in primary care,^10^ GPs may not be confident in their diagnosis and treatment, resulting in referrals to urology specialists that are potentially avoidable.

To address clinical uncertainty and variations in practice, in 2010 the National Institute for Health and Care Excellence published guidance (revised 2015) on the assessment and management of male LUTS in primary care.^11^ The guidance indicates that males with uncomplicated LUTS should be referred to a specialist only where LUTS are bothersome, and after conservative management and drug treatment have been found ineffective.^11^ However, incident referrals to UK urology outpatients for males aged ≥16 years increased by around 20% from 2014/2015 to 2018/2019.^12^^,^^13^

Research relating to outcomes for patients with LUTS has tended to focus on comparison of specific interventions rather than patient satisfaction with their care as a whole, an area that has been little explored.^14^ Limited evidence indicates that treatment is frequently ineffective from a patient perspective. For example, in one observational study of males being treated for LUTS in primary care,^15^ around half of participants reported unsatisfactory outcomes, such as persisting or worsening symptoms. A qualitative interview study also found that most participants experienced no or only partial relief of their LUTS after consulting a clinician, although they generally reported satisfaction with the care they received.^14^

**Table:** How this fits in

Lower urinary tract symptoms (LUTS) in males can usually be treated effectively in primary care; however, referrals to urology services are increasing. This study explores in detail the experiences of GPs and patients in relation to the management of LUTS in primary care. Difficulty establishing causes and differentiating symptoms were identified as key challenges; therefore, treatment was often a process of trial and error, and no patient’s symptoms were completely resolved. A diagnostic tool for use by GPs, together with greater exploration of non-pharmacological treatment approaches, could support effective management of LUTS in primary care settings.

Despite the challenges of managing LUTS in primary care, little research has explored in depth the perspectives of GPs themselves or investigated the experiences of patients. This qualitative interview study aimed to explore GPs’ experiences of diagnosing and managing LUTS, together with patients’ experiences of and preferences for treatment of LUTS in primary care.

## METHOD

### Study design and setting

Telephone interviews were conducted with GPs and patients from GP practices involved in the PriMUS (Primary care Management of lower Urinary tract Symptoms in men: development and validation of a diagnostic and clinical decision support tool) study^16^ across three UK regions: Newcastle upon Tyne, Bristol, and South Wales. PriMUS is a prospective diagnostic accuracy study aimed at developing a decision tool to help GPs more accurately diagnose and manage LUTS in males. PriMUS participants are males aged ≥16 years presenting to their GP with a complaint of ≥1 bothersome LUTS, including those receiving treatment (see the PriMUS study protocol^16^ for full inclusion and exclusion criteria). PriMUS participants receive a series of index tests and invasive urodynamics, conducted in primary care settings by trained research nurses.

### Sampling and recruitment

A purposive sampling strategy was used to ensure representation of patients and GPs from the three study regions, and to include patients who opted not to take part in the main PriMUS study, in addition to main study participants.

Patients participating in the main PriMUS study were invited to take part via telephone or email, while those who chose not to participate in the main study were given brief information about the interview study by the recruiting clinician. If they expressed an interest in the interview study, patients were provided with an information sheet, a prepaid envelope, and a consent form that they were asked to return to the first author if they wished to take part. Patients were contacted by this same author after they had undergone all PriMUS study procedures or had decided not to take part in the main study, and the interview was arranged at a mutually convenient time. A 10 GBP voucher was offered to patient participants to thank them for their participation. GPs involved in the PriMUS study were invited via email to take part in an interview. If they agreed to take part after reading the study information sheet, an interview was arranged at a mutually convenient time. Informed consent was obtained verbally at the start of each interview and was audio-recorded.

### Data collection

Semi-structured interview topic guides were developed specifically to address the aims of the study, drafted by the study qualitative researchers (the first and second authors), and revised following feedback from clinicians and patient representatives on the PriMUS study management group. Patient interview topic guides aimed to explore patients’ experiences of LUTS, their decision to visit the GP about their symptoms, satisfaction with treatment, preferences for treatment in primary versus secondary care, and preferences for involvement in treatment decisions. GP interview topic guides aimed to explore experiences of identifying causes and treatment options, and managing LUTS in general practice. GP interviews were designed to be relatively brief to encourage participation.

Interviews were conducted from May 2018 to January 2019. All GP interviews and most patient interviews were conducted by the first author, a qualitative researcher experienced in health research. A sample of patient interviews was conducted by medical students (authors 4–6), supervised by the first author. The interviewers were previously unknown to participants and had no specialist clinical knowledge of LUTS. Interviews were audio-recorded with the permission of participants and transcribed verbatim. Data collection continued until no new themes emerged from the data.

### Data analysis

Framework analysis was used.^17^ All interview transcripts were read by the first author, who developed a coding framework based on emerging themes and topics covered in the interview guides. NVivo (version 11) was used to organise data into the themes identified in the framework. Ten per cent of interview transcripts were independently coded by a second qualitative researcher (the second author) to ensure consistency in the way the codes were applied. The first author then compiled separate tables for GPs and patients to summarise the experience of each interviewee in relation to each of the identified themes. The two qualitative researchers met to discuss these data tables and identify and agree on key themes in relation to the research aim.

## RESULTS

In total, 25 male patients and 11 GPs from 20 GP practices across Newcastle upon Tyne, South Wales, and Bristol participated in an interview (see Table 1). Patients were aged between 48 and 85 years (mean age 67 years). By the 6-month follow-up point of the PriMUS study, one interviewed patient had been referred to secondary care for further investigation and subsequent treatment. GP interviews lasted between 10 and 22 min (mean 17 min); patient interviews lasted between 8 and 44 min (mean 23 min).

**Table 1.** Participant characteristics

**Characteristic**	***n***
**Patients *(n* = 25)**	

Participant in main study	
Yes	22
No	3
Geographical region	
Newcastle upon Tyne	10
South Wales	9
Bristol	6
Age group, years^a^	
46–55	3
56–65	6
66–75	8
76–85	5
IPSS^a^	
1–7 (mild)	3
8–19 (moderate)	12
20–35 (severe)	7

**GPs (*n* = 11)**	
Sex	
Male	7
Female	4
Geographical region	
Newcastle upon Tyne	5
South Wales	4
Bristol	2
Years on GP register	
0–5	3
6–10	4
≥11	4

a *Age and IPSS data were not recorded for patients who did not participate in the main PriMUS study.^16^ IPSS = International Prostate Symptom Score.*

Interview data were organised into four main themes: unresolved symptoms, preference for primary care, satisfaction with involvement in decision making, and challenges of managing LUTS in primary care. Quotes in this section are labelled with each participant’s unique identification number, prefaced by ‘P’ for patients and ‘GP’ for GPs.

### Unresolved symptoms

None of the patients interviewed reported that their symptoms had been fully resolved following their visit(s) to the GP. Most had received no treatment for their LUTS (treatment here refers to prescribed medication as opposed to lifestyle advice or watchful waiting). Although in some cases this was because tests were still in progress, for other patients tests had been completed and no course of treatment had been prescribed. Patients believed this was because nothing could be done or because symptoms were normal for their age. Despite ongoing symptoms, some expressed satisfaction with GP consultations, as they had been reassured that there was no serious underlying cause such as prostate cancer:

*‘* [When my symptoms] *first started happening obviously you read about things … the cancer thing … I was worried about that, and I went to see the doctor. And he took the blood tests … and everything, which came back OK, you know. So … that’s a relief, takes a lot off my mind … I just accept the fact now it’s part of growing older I suppose.’*

(P2015)

*‘It seems there was nothing* [the doctor] *could have done, I don’t think. Because I’m not on any treatment, he didn’t give me anything for it.’*

(P3103)

Of those who had received treatment for their LUTS, some said this had no effect, while others reported that it had made some difference but not completely resolved bothersome symptoms:

*‘I took* [tablets] *for 12 months, and I saw no difference whatsoever, so I stopped taking them.’*

(P1002)

*‘I had to get up about five or six times a night … it was really getting me down … They changed my medication to … tamsulosin … and it, it just sort of keeps me down to about three times a night but it’s still at least three times a night.’*

(P1023)

Intolerable side effects of medication were reported that had led, in most cases where they occurred, to treatment being discontinued or substituted. Several patients had tried multiple treatment options:

*‘* [The doctor] *gave me some tablets, because of the frequency of getting up in the night. But I … wasn’t very good with those, they swelled my ankles and I felt a bit dizzy with them. So I said to him, “I’m not taking them because I’m not very happy with it.” … So I’ve just put up with it, more or less. I kind of control it by not drinking. Which is not the best thing to do, of course, because you get dehydrated.’*

(P3133)

*‘I have had quite a difficult time with the tablets I was originally given … it knocked me for six, I never felt so awful taking a tablet that was supposed to help … I have been given some others, but I had to come off them, because I was so muzzy headed, weak …’*

(P202)

Some patients were dissatisfied with the way their LUTS had been managed, because of the feeling that their symptoms had not been adequately explained or treated:

*‘*[The doctor] *gave me some tablets … and I tried them … they give me a lot of nightmares and one thing and another. So I went back to* [the doctors] *and … he gave me the name of some herbal remedy to try and he said it’s sort of due to my age … Which I was a bit disappointed about … I’m only fifty-six … so it’s not exactly old.’*

(P3110)

Patients did not all feel that their LUTS had been thoroughly examined, and appreciated the opportunity to have a thorough diagnostic test as part of the PriMUS study.

### Preference for primary care

Patients expressed a preference for having their LUTS treated in primary rather than secondary care. This tended to be because visiting the GP was more convenient, either because of the locality of the GP practice or shorter waiting times:

*‘You go to the doctors, you can see the doctor the same day or the next day. You go to the hospital … you are going to wait for hours on end, because they are so busy. I wouldn’t go to the hospital unless I felt I had a serious condition, that my GP would have referred me to the hospital* [for] *anyway.’*

(P1002)

Patients also commented that they felt more comfortable at their GP surgery as staff were familiar and knew their history. This meant that they were more relaxed about having potentially invasive tests. Some emphasised that they felt completely confident in their GP to provide their care:

*‘For me, going to my GP makes it so much easier because he knows my history. Whereas in a hospital you start again … and then you get passed on to someone else and it just goes on and on and on …* [my doctor] *sort of remembered me, so that was so much easier than being with another, you know, strange doctor.’*

(P2002)

*‘You feel comfortable* [at the GP practice] *, it’s a more comfortable surrounding and all. You just go in there just in the room, one, one person you know … I would be totally confident in* [my GP to run the tests that were done in hospital]. *Everything that was done there, if it could be done at the GPs it would be brilliant.’*

(P2015)

Patients generally felt there would be no benefit in attending hospital for the diagnosis and treatment of their LUTS. They believed secondary care would only be useful in certain circumstances, for example, if specialist advice or equipment were needed for invasive and complex procedures, or in an emergency:

*‘I suppose in a hospital, you’re more likely to get specialist advice …* [But going to hospital is] *something to be endured, it’s not really somewhere you want to go is it?’*

(P2004)

### Satisfaction with involvement in decision making

Patients stated that they would be more likely to adhere to recommended treatment for their LUTS if they felt involved in decision making. Levels of involvement varied, with some patients following doctors’ recommendations, others being informed of the reasons for their treatment, and others fully involved in decision making. Despite this variation, all were satisfied with their level of involvement:

*‘I think the more you understand … why the treatment is there, what it’s aiming to do, I think it’s easier to stick to it … In fairness … all the treatment I’ve had over the years, I’ve always felt my doctors have said, look this is why we’re doing this … so I’ve always felt that I’ve been kept informed.’*

(P1058)

Some patients reported that they would prefer treatment decisions to be made solely by their doctor, although it was acknowledged that the decision as to whether to proceed with medication was their own:

*‘I was quite happy for* [my GP] *to just organise it all and get on with it.’*

(P2001)

*‘The way I see it, the doctors, they’re the professionals. So basically, you go along with their advice … I’d always listen to what they say, but at the end of the day the decision’s with yourself isn’t it, you know. But I always take, try to take advice.’*

(P2015)

### Challenges of managing LUTS in primary care

**Figure 1.**
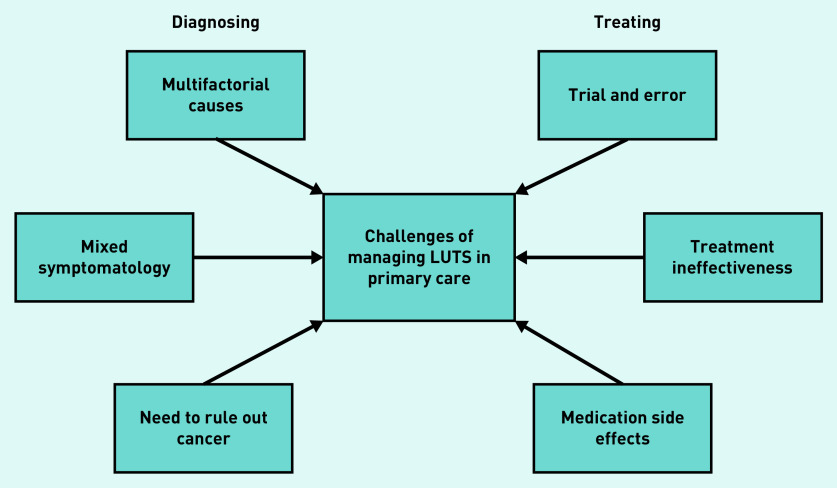
*Challenges of managing lower urinary tract symptoms (LUTS) in primary care.*

Challenges identified by GPs could be separated into those relating to diagnosis and those relating to treatment (see Figure 1). In terms of diagnosis, a key challenge was that the cause of LUTS can be multifactorial, and therefore difficult to establish. Compounding this, GPs reported that patients often present with mixed symptoms (that is, both voiding and storage symptoms), making treatment decisions more complex. It was acknowledged that GPs may be less likely to identify uncommon causes of LUTS, such as urethral stricture. Diagnoses tended to be largely based on patient history, because of a lack of diagnostic tools available in primary care. Reliance on patient reporting could be problematic, however, for example, with difficulties obtaining accurate reports of patients’ fluid intake:

*‘The cause of lower tract infections can be multifactorial … that is one of the challenges, so* [are symptoms] *due to increased fluid intake, is it due to caffeine? You do have to take quite a detailed history and sometimes patients don’t think about* [what contains caffeine] *or, you know, “I don’t really drink”,* [but they have] *two glasses of whisky at night time.’*

(GP401)

*‘Sometimes you meet men where it’s very clear what the cause of their LUTS is, but in the majority it’s, it comes across as a mixed picture of urgency symptoms, voiding symptoms and you know, that can be difficult, and what you end up often doing is picking what you think is most likely and trying a treatment and then reviewing the patient.’*

(GP502)

GPs highlighted the difficulty of differentiating between prostate and bladder symptoms to eliminate the possibility of prostate cancer. Greater awareness among patients due to public health campaigns meant that this was a particular concern and often the reason patients in this study decided to visit their GP. As LUTS are common among males aged ≥40 years, it was considered that increased awareness of prostate cancer symptoms may result in undue concern:

*‘A good thing is greater awareness* [of] *prostate cancer of course … but it can come with problems as well, because … the majority of men are going to have lower urinary tract symptoms at some point in their life, especially as they get older, and then I suppose a lot of men in that situation, their main concern is prostate cancer. So … if there was a really good prostate screening tool … that would be really helpful.’*

(GP405)

Because of difficulties diagnosing LUTS, treatment was described as a process of trial and error. A lack of guidance on treatment options was reported, resulting in uncertainty, particularly where symptoms were mixed:

*‘It is a bit of a stab in the dark very often … you kind of take* [the patient’s] *history and you try some drugs, and you try some other drugs.’*

(GP604)

*‘I think sometimes it is just trying a few different medications and seeing which helps … It’s not exact science … we try trial and error.’*

(GP401)

GPs identified that available treatments for LUTS were often ineffective, and expressed concerns about the possibility of side effects, particularly for older patients. As reported by patients, this resulted in medication being discontinued in some cases and LUTS remaining untreated. GPs suggested the less harmful alternative of non-pharmacological approaches was not always considered:

*‘The treatments I must say aren’t the best, certainly we have quite a lot of unsuccessful … medication trials. Now whether that’s due to patient compliance, side effects or, you know, the expectations. So we are often changing medications over to an alternative one, and sometimes we just give up in the end.’*

(GP403)

*‘I guess the problem … is particularly in elderly men, a lot of these treatments have side effects … there’s lots of other non-pharmacological treatments … to give the patient that might be safer and more effective for them.’*

(GP502)

## DISCUSSION

### Summary

The mixed symptomatology and multifactorial causes of LUTS made differentiating symptoms (including ruling out the possibility of cancer) and deciding on an appropriate course of treatment challenging for GPs. Therefore, treating LUTS was a process of trial and error, with some patients receiving multiple treatments. None of the patients interviewed felt that their symptoms had been fully resolved. This appeared to because of a range of factors, including difficulty in establishing the causes of LUTS, and GPs were particularly concerned about the relative ineffectiveness of pharmacological treatments for LUTS and the possibility of side effects, particularly for older patients. Patients themselves reported intolerable side effects of medication, which had led to the cessation of treatment in some cases. GPs suggested that non-pharmacological approaches to the treatment of LUTS should be given greater consideration.

GPs and patients highlighted that males presenting with LUTS were often seeking reassurance that their symptoms were not an indication of prostate cancer, owing to increased public awareness of the disease. Patients who attended their GP for this reason appeared to be satisfied with their consultations despite their LUTS not being effectively treated. GPs emphasised the difficulty of differentiating between prostate and bladder symptoms so as to eliminate the possibility of prostate cancer.

Patients reported that they would prefer their symptoms to be managed in primary care where possible, citing the convenience of greater accessibility and shorter waiting times, together with confidence in clinicians familiar with their history. Patients’ preferences for involvement in decisions about their treatment varied, with most wanting to be involved in decision making, while some preferred decisions to be made by their GPs.

### Strengths and limitations

The key strength of this study is the inclusion of the perspectives of both patients and GPs, enabling an in-depth examination of the management of LUTS in primary care. Interviewees represented three diverse regions of the UK, enhancing the generalisability of findings. A further strength is the collaborative development of the research by researchers, clinicians, and patient representatives on the study management group. This helped to ensure participant-facing materials were appropriate and that the interview topic guides encompassed issues considered important by key stakeholders.

Although GP interviews were brief, the research aim was tightly focused, and participants had highly specific experience in relation to the topic being explored; these factors enable information power to be achieved with a smaller sample.^18^ Consistent with evidence on thematic saturation,^19^ most key themes were present within the first three GP interviews.

All GPs interviewed were based in research-active practices that had opted to take part in a study focusing on the diagnosis of LUTS, and therefore may not be representative of primary care settings across the UK. Nevertheless, a number of key challenges in the management of LUTS were identified that have implications for practice in general.

The GP interviews were conducted by a non-clinical researcher; this not only provided an independent perspective but may also have affected the way in which GPs discussed challenges in their practice.

### Comparison with existing literature

To the authors’ knowledge, no studies have previously explored in depth the perspectives of GPs in relation to the management of LUTS. This study has enabled the identification of key challenges in diagnosing and treating LUTS in primary care. Consistent with research highlighting the complexity of diagnosing LUTS in males,^9^ GPs reported that difficulty differentiating symptoms and considering multifactorial causes made it challenging to decide on an appropriate treatment.

The present findings build on limited research into patient satisfaction with the management of LUTS in primary care. In line with previous studies,^14^^,^^15^ patients reported that their symptoms had not been fully resolved. Contrary to research demonstrating the general efficacy of LUTS medication,^20^ GPs and patients in this study suggested that treatments were not fully effective. Yet, as previously found,^14^ patients were generally satisfied with their care. Further exploration of this apparent contradiction revealed that patients were commonly concerned that their symptoms could be indicative of prostate cancer and were therefore satisfied with the reassurance provided by their GP, despite their LUTS not being treated effectively. Public health campaigns had not only raised awareness of prostate cancer, but also had the effect of increasing potentially undue concern among patients. Research suggests that surveillance for (over)diagnosed prostate cancer can have negative psychological consequences such as feelings of uncertainty and powerlessness.^21^

Consistent with existing findings,^7^ over half of patients in this study had received no prior treatment for their LUTS. As reported previously,^14^ patients perceived this to be because their symptoms were age related. Concerns expressed by GPs relating to effectiveness of treatments for LUTS and the high incidence of side effects in older males may also partially explain the lack of treatment prescribed in some cases.

To the authors’ knowledge, this is the first reported study to examine patient preferences for management of LUTS. Studies relating to other health conditions have reported mixed findings regarding preferences for primary or secondary care. For example, cancer survivors tend to prefer specialist follow-up,^22^^–^^24^ whereas limited research indicates that patients with mental illness may prefer to access their own GP practice.^25^^,^^26^ A survey relating to epilepsy services found that, while younger patients and those with severe epilepsy preferred secondary care, older patients preferred to receive care from their GP.^27^ Patients in this study, who tended to be older (mean age 67 years), expressed a strong preference for having their LUTS treated in primary care. Their reasoning aligned with the three types of continuity of care proposed by Reid *et al*:^28^ informational continuity (GPs had access to a holistic clinical record system); relational continuity (patients had confidence in their GP and felt more comfortable with familiar staff and surroundings); and management continuity (patients could access timely and convenient care via their GP practice).

### Implications for research and practice

The present study findings emphasise the importance of LUTS being managed in primary care where possible, as in addition to cost savings and reduced waiting times, this is a more accessible option for patients, who tend to be more comfortable and confident being treated by familiar clinicians. To address the identified challenges of managing LUTS in primary care, prostate cancer risk management^29^ or LUTS diagnostic tools would be helpful. It appears that bothersome LUTS are in some cases dismissed as a normal part of the ageing process; ensuring that such symptoms are managed well is essential to enhance patients’ quality of life. Further research could explore how the belief that symptoms are age related impacts on treatment expectations among patients, and also the role of comorbidity and polypharmacy in influencing readiness to seek medical help for these symptoms.

Considering the reported lack of effectiveness and intolerable side effects of some LUTS medication, greater exploration of non-pharmacological treatment would be beneficial. A study of urology outpatients^30^ has shown promising results for the effectiveness of sessions promoting the self-management of LUTS; however, the authors suggest that a large randomised controlled trial is needed to confirm findings.

Patients in this study indicated a strong preference for their symptoms to be managed in primary care. Given that preference for primary versus secondary care appears to vary between different patient groups,^22^^–^^27^ it would be interesting to further explore the effect of patient characteristics on treatment preferences.
